# Detection of Capripoxvirus DNA Using a Field‐Ready Nucleic Acid Extraction and Real‐Time PCR Platform

**DOI:** 10.1111/tbed.12447

**Published:** 2015-11-25

**Authors:** B. Armson, V. L. Fowler, E. S. M. Tuppurainen, E. L. A. Howson, M. Madi, R. Sallu, C. J. Kasanga, C. Pearson, J. Wood, P. Martin, V. Mioulet, D. P. King

**Affiliations:** ^1^ The Pirbright InstituteSurreyUK; ^2^ Institute of Biodiversity, Animal Health and Comparative MedicineUniversity of GlasgowGlasgowUK; ^3^ Tanzania Veterinary Laboratory Agency (TVLA)Dar‐es‐SalaamTanzania; ^4^ Faculty of Veterinary MedicineSokoine University of AgricultureMorogoroTanzania; ^5^ Enigma DiagnosticsDSTL Porton DownSalisburyUK

**Keywords:** virus, diagnostics, disease control, disease‐freedom, emerging diseases, capripoxviruses

## Abstract

Capripoxviruses, comprising sheep pox virus, goat pox virus and lumpy skin disease virus cause serious diseases of domesticated ruminants, notifiable to The World Organization for Animal Health. This report describes the evaluation of a mobile diagnostic system (Enigma Field Laboratory) that performs automated sequential steps for nucleic acid extraction and real‐time PCR to detect capripoxvirus DNA within laboratory and endemic field settings. To prepare stable reagents that could be deployed into field settings, lyophilized reagents were used that employed an established diagnostic PCR assay. These stabilized reagents demonstrated an analytical sensitivity that was equivalent, or greater than the established laboratory‐based PCR test which utilizes wet reagents, and the limit of detection for the complete assay pipeline was approximately one log_10_ more sensitive than the laboratory‐based PCR assay. Concordant results were generated when the mobile PCR system was compared to the laboratory‐based PCR using samples collected from Africa, Asia and Europe (*n* = 10) and experimental studies (*n* = 9) representing clinical cases of sheep pox, goat pox and lumpy skin disease. Furthermore, this mobile assay reported positive results *in situ* using specimens that were collected from a dairy cow in Morogoro, Tanzania, which was exhibiting clinical signs of lumpy skin disease. These data support the use of mobile PCR systems for the rapid and sensitive detection of capripoxvirus DNA in endemic field settings.

Capripoxviruses (CaPVs) cause serious pox diseases of domesticated ruminants (Carn, 1993). Comprising sheep pox virus (SPPV), goat pox virus (GTPV) and lumpy skin disease virus (LSDV), they are large, complex, double‐stranded DNA viruses within the genus *Capripoxvirus*, subfamily *Chordopoxvirinae*, family *Poxviridae* (Buller et al., 2005). SPPV and GTPV are normally restricted to Asia and North Africa, although clinical cases of sheep pox have also been detected in Europe (Mangana et al., 2008), and recently in Bulgaria and Greece (during 2013). Lumpy skin disease (LSD) occurs across Africa, and in recent years, LSDV has also been found in several countries of the Middle East (Tuppurainen and Oura, 2012), including Turkey where more than 236 outbreaks have occurred since 2013 (ProMed 20130831.1915595).

The World Organization for Animal Health (OIE) classifies CaPVs as notifiable disease agents, and molecular diagnostic tests play an important role in monitoring the spread of these viruses in susceptible livestock. A range of conventional agarose‐gel‐based polymerase chain reaction (PCR) assays (Ireland and Binepal, 1998; Heine et al., 1999; Markoulatos et al., 2000; Tuppurainen et al., 2005; Zheng et al., 2007), or real‐time PCR assays (Balinsky et al., 2008; Bowden et al., 2008; Stubbs et al., 2012) are used in diagnostic laboratories. However, poorly equipped laboratories often face difficulties accessing these molecular techniques (particularly real‐time PCR) that are reliant upon expensive and relatively fragile equipment. In particular, the ability to perform nucleic acid‐based tests such as PCR in field settings has proven to be a challenging goal largely due to the reliance upon pre‐processing of samples (nucleic acid extraction), the lack of stable reagents that are suitable for use in environments where it is not possible to maintain a cold chain (King et al., 2008) and the cost of the field equipment. The Enigma Field Laboratory (FL) is a hardware platform which undertakes nucleic acid extraction, PCR thermocycling and analysis of data without user intervention, which has been applied for the detection of other notifiable diseases such as foot‐and‐mouth disease (Madi et al., 2012). The aim of this study was to optimize and evaluate a mobile PCR platform for the simple detection of CaPV DNA.

This study utilized the real‐time PCR primers, probes, master mixes and cycling conditions that have been previously described (Bowden et al., 2008). Pilot studies were undertaken to assess the performance of newly developed lyophilized PCR reagents that were prepared and assembled into assay cartridges by Enigma Diagnostics (Salisbury, UK). A decimal dilution series (Neat to 10^−10^) of DNA prepared from an LSDV isolate (Israel LSD‐07 POX‐V1‐07‐08, isolated from naturally infected cattle in 2007) was prepared in nuclease‐free water containing carrier RNA (1 *μ*g ml^−1^). In these experiments, 5 *μ*l of each dilution was mixed with 20 *μ*l of nuclease‐free water and this was used to re‐suspend the lyophilized reagent pellets prepared by Enigma Diagnostics. This 25 *μ*l suspension was then transferred into a 96‐well PCR plate. For the conventional wet reagents, 2 *μ*l of DNA was added to 18 *μ*l of diagnostic assay mastermix (Bowden et al., 2008) prior to transfer into 96‐well PCR plate. This initial laboratory validation of lyophilized reagents was carried out on the Mx3005P quantitative PCR machine (Stratagene). Parallel testing demonstrated an improved analytical sensitivity of one log_10_ for the lyophilized reagents when compared to the reference test (Fig. 1a). This one log_10_ increase in analytical sensitivity was maintained when the new lyophilized assay was applied to a decimal dilution series (10^−1^–10^−10^) of Israel LSD‐07 POX‐V1‐07‐08 virus prepared in 10% w/v homogenized cattle skin suspensions and run in full, including DNA extraction on the Enigma FL (Fig. 1b). For the above comparison, one aliquot per dilution was extracted on the MagNA Pure LC Robot (Roche) using the Total Nucleic Acid Isolation kit (Roche) following manufacturer guidelines, followed by real‐time PCR performed using wet reagents assayed on the Mx3005P quantitative PCR machine (Stratagene) (reference test). The extraction and real‐time PCR for the second aliquot (0.5 ml) was performed in a complete automated cycle on the Enigma FL.

**Figure 1:**
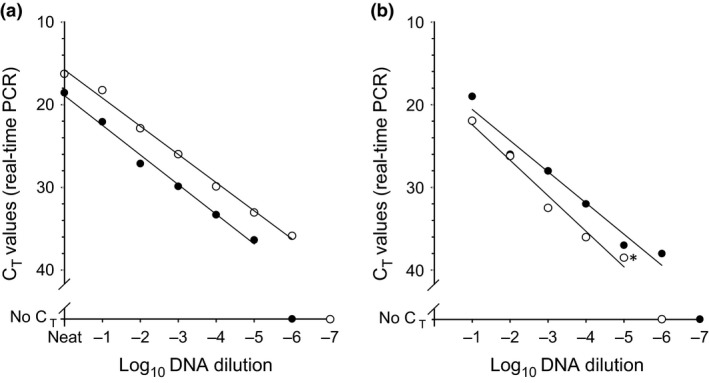
Comparative analytical sensitivity of the lyophilized PCR assay used to detect CaPV DNA. (a) A decimal dilution series of CaPV DNA (isolate Israel LSD‐07 POX‐V1‐07‐08) tested using wet reagents (●) and lyophilized reagents (○) with a laboratory‐based PCR machine (Mx3005P, Stratagene). Points represent mean C_T_ from duplicate determinations where the maximum C_T_ range of duplicates was 1.53. (b) Comparison of a decimal dilution series of a CaPV isolate DNA (Israel LSD‐07 POX‐V1‐07‐08) spiked into skin suspensions assayed using wet PCR reagents (○, Mx3005P) compared to lyophilized PCR reagents and nucleic acid extraction employed on the Enigma FL (●). Points represent mean C_T_ from duplicate determinations for the wet PCR assay (* data point represents a single where the duplicate sample generated a no C_T_ result), while only single values are shown for the samples tested on the Enigma FL.

The suitability of this assay to detect CaPV DNA in clinical samples was evaluated using archived field and experimental samples held at the OIE Reference Laboratory for LSDV and GPV/SPV (The Pirbright Institute, UK) and the National Veterinary Reference Laboratory in Tanzania (Tanzania Veterinary Laboratories Agency‐TVLA). Fourteen clinical samples (Table 1), comprising nine from two experimentally infected animals where cattle had been infected with LSDV Neethling strain (VN83 and VN84), and five from field samples submitted to The Pirbright Institute were used. Five additional skin scrapings from the TVLA archive were analysed within East African laboratory settings. For each sample, one duplicate was extracted and assayed using the established reference test (Bowden et al., 2008) whilst for the second duplicate the extraction and real‐time PCR was performed on the Enigma FL. There was complete concordance between positive results (*n* = 19) and negative results (*n* = 3) generated on the Enigma FL and the standard laboratory pipelines (Table 1). Opportunistic testing of samples collected from a Holstein–Friesian cross‐dairy cow on a small holder farm in Morogoro, Tanzania, displaying clinical signs of LSD (Fig. 2) was also undertaken. Two samples comprising EDTA blood and skin scrapings were tested; skin scrapings were processed using a field‐based tissue processing kit (Svanodip^®^ Ag extraction kit; prior to being added into the Engima FL sample loading chamber, whilst EDTA blood was added directly to the sample chamber). Both specimens were positive for CaPV DNA using the Enigma FL. It should be noted that the current diagnostic reference test (Bowden et al., 2008) utilizes a conservative cut‐off C_T_ value of <37 to define a positive result. However, for this study, all C_T_ values are reported because amplification of CaPV in animals with late infection may generate weak values that would be missed with a cut‐off of 37. Suitable negative controls were also included in the data set to confirm the absences of any false amplification.

**Table 1:** Performance of the lyophilized PCR system (Enigma FL) compared to the laboratory‐based PCR pipeline (Reference Test) using clinical samples collected from field cases and experimental infection studies

	Sample ID	Species	Sample type	Enigma FL C_T_	Reference test C_T_
Experimental infection	VN83 11DPI	Cattle	Blood	33	35.74
	VN83 14DPI	Cattle	Ocular	39	35.93
	VN83 16DPI	Cattle	Saliva	43	33.38
	VN83 25DPI	Cattle	Nasal	36	32.90
	VN83 28DPI	Cattle	Saliva	39	33.83
	VN83 37DPI	Cattle	Scabs	18	15.71
	VN84 12DPI	Cattle	Blood	34	34.71
	VN84 14DPI	Cattle	Ocular	38	34.86
	VN84 22DPI	Cattle	Scabs	17	16.91
Field samples	BUL/V713‐1 2013	Sheep	Blood	34	29.96
	BUL/V713‐3 2013	Sheep	Blood	33	28.93
	MON/V107‐1 2007	Sheep	Blood	16	11.50
	MON/V107‐3 2007	Sheep	Scabs	16	12.53
	VIET/21030‐2 2005	Goat	Scabs	22	18.99
	TAN/TVLA 1 GRS^a^	Cattle	Scabs	16	16.12
	TAN/TVLA 2 NS^a^	Cattle	Scabs	31	31.23
	TAN/TVLA 3 MR^a^	Cattle	Scabs	26	26.34
	TAN/TVLA 4 MS^a^	Cattle	Scabs	18	18.18
	TAN/TVLA 5 GM^a^	Cattle	Scabs	34	34.14
	TAN/Morogoro^b^	Cattle	Blood	35	Not tested
	TAN/Morogoro^b^	Cattle	Scabs	19	Not tested
Control	UKG Negative	Cattle	Blood	No C_T_	No C_T_
	UKG Negative	Sheep	Blood	No C_T_	No C_T_
	UKG Negative	Cattle	Skin	No C_T_	No C_T_

a Five samples were skin nodule scrapings from separate cattle with clinical LSD from Mwika Village, Kilimanjaro, Tanzania.

b Field samples from Morogoro, Tanzania, collected and analysed *in situ*.

**Figure 2:**
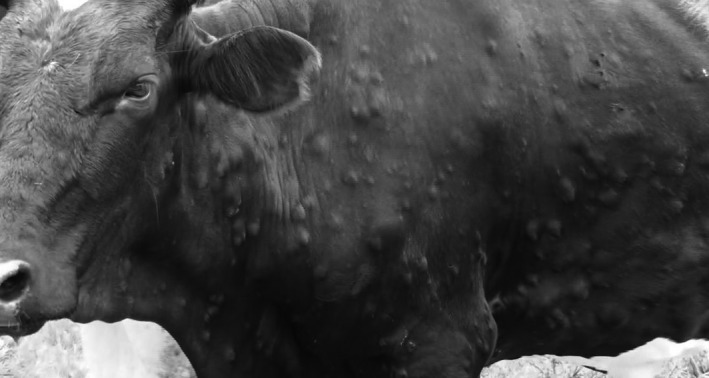
Holstein–Friesian cross‐dairy cow displaying clinical signs of lumpy skin disease (LSD) on a farm in Morogoro, Tanzania, from which field samples (EDTA blood and skin nodule scrapings) were tested.

These data show that a mobile PCR platform can rapidly detect CaPVs from suspect cases, within 60 minutes of sample collection, offering a sensitive molecular technology that can be deployed into field settings. Furthermore, the comparative data for the lyophilized reagents demonstrate that it is possible to generate a stabilized assay with equivalent (or better) performance compared with the wet‐assay format. This current assay format represents the results of a collaborative research project that was undertaken to highlight the potential of these technologies for diagnostic use. Future validation to include a greater sample data set would increase confidence in the test and could be combined with optimization of the DNA extraction steps to bring its performance in line with that of a standard laboratory extraction robot. Further availability of this particular assay (via commercial sources), as well as other tests that might also exploit this format, will be dependent upon demand and interest from customers.

## Conflict of Interest

James Wood and Paul Martin are employees of Enigma Diagnostics. All laboratory work and evaluation of the equipment was undertaken by staff from The Pirbright Institute, Sokoine University of Agriculture and TVLA, and no financial support was provided from Enigma Diagnostics to The Pirbright Institute to conduct this study.
